# Does the Addition of Pain Neurophysiology Education to a Therapeutic Exercise Program Improve Physical Function in Women with Fibromyalgia Syndrome? Secondary Analysis of a Randomized Controlled Trial

**DOI:** 10.3390/jcm10112518

**Published:** 2021-06-07

**Authors:** Luis Ceballos-Laita, María Teresa Mingo-Gómez, Elena Estébanez-de-Miguel, Elena Bueno-Gracia, Francisto José Navas-Cámara, Zoraida Verde-Rello, Ana Fernández-Araque, Sandra Jiménez-del-Barrio

**Affiliations:** 1 Department of Surgery, Ophthalmology and Physiotherapy, Faculty of Health Sciences, University of Valladolid, c/Universidad s/n, 42004 Soria, Spain; luis.ceballos@uva.es (L.C.-L.); tmingo@cir.uva.es (M.T.M.-G.); fjnavas@bio.uva.es (F.J.N.-C.); 2 Department of Physiatrist and Nursey, Faculty of Health Sciences, University of Zaragoza, c/Domingo Miral s/n, 50010 Zaragoza, Spain; elesteba@unizar.es (E.E.-d.M.); ebueno@unizar.es (E.B.-G.); 3 Department of Biochemistry, Molecular Biology and Physiology, Faculty of Health Sciences, University of Valladolid, c/Universidad s/n, 42004 Soria, Spain; zoraida.verde@uva.es; 4 Department of Nursey, Faculty of Health Sciences, University of Valladolid, c/Universidad s/n, 42004 Soria, Spain; anamaria.fernandez@uva.es

**Keywords:** fibromyalgia, exercise, patient education, functionality, fatigue

## Abstract

Therapeutic exercise (TE) is one of the most investigated approaches for the management of FMS. Pain neurophysiology education (PNE) helps toward understanding the pain condition, leading to maladaptive pain cognitions and coping strategies in patients with chronic pain. Our study aimed to assess the effects of therapeutic exercise and pain neurophysiology education versus TE in isolation on fatigue, sleep disturbances, and physical function in the short term and at three months of follow-up in women with fibromyalgia syndrome (FMS). A single-blind randomized controlled trial was carried out. A total of 32 women with FMS referred from medical doctors and fibromyalgia association were randomized in 2 groups: PNE + TE group or TE group. Fatigue and sleep disturbances (Visual Analog Score) and physical function (Senior Fitness Test) were assessed before, after intervention, and at three months of follow-up. Significant improvements were achieved in the Timed Up and Go test (*p* = 0.042) and Arm Curl test (*p* = 0.043) after intervention and on handgrip in the non-dominant side at three months of follow-up (*p =* 0.036) on the PNE + TE group. No between-groups differences were found for fatigue, sleep disturbances, and the rest of test included in the Senior Fitness Test. In conclusion, these results suggest that PNE + TE appears to be more effective than TE in isolation for the improvement of physical function (Timed Up and Go test and Arm Curl test) in women with FMS in the short term.

## 1. Introduction

Fibromyalgia syndrome (FMS) is a complex multidimensional disease characterized by widespread pain associated with other non-pain symptoms, such as fatigue and sleep disturbances [1,2]. The wide variety of symptoms reported by patients with FMS contributes to a physical deconditioning, which affects daily living activities [3].

People with FMS often present low cardiovascular fitness [4], muscle strength, and muscle endurance [5,6]. This physical deconditioning is closely linked to other symptoms, such as fatigue [7]. Fatigue increases with activity, which often makes patients with FMS intolerant to physical activity and means they tend to have a sedentary lifestyle [8], thus potentially increasing their risk of additional morbidity [9,10,11].

Therapeutic exercise (TE) is one of the most investigated approaches for the management of FMS. Aerobic exercise, strengthening, and their combination have shown similar effects in improving fatigue, sleep disturbances, and physical function [12,13,14,15,16]. The effects of patient education have also been investigated in patients with chronic conditions. Pain neurophysiology education (PNE) helps toward understanding the pain condition, leading to improvements in patients’ management of maladaptive pain cognitions and in coping strategies among patients with chronic pain. PNE focuses on thoughts and attitudes that could be strong predictors of persistent disability [17]. However, a recent systematic review reported no effects on physical function in patients with FMS following patient education in isolation [18].

The most recent clinical guidelines recommend the combination of TE and patient education for FMS [19]. The addition of PNE to TE could produce beneficial effects on physical function in patients with FMS. To the best of our knowledge, there is a lack of evidence regarding the effects of TE (aerobic exercise and strengthening) combined with PNE on fatigue, sleep disturbances, and physical function in patients with FMS.

Thus, the aim of the current study was to compare the effects of PNE + TE compared to TE in isolation on fatigue, sleep disturbances, and physical function in women with FMS.

## 2. Material and Methods

### 2.1. Study Design

The data presented in this article represent a secondary analysis of a randomized single-blind controlled clinical trial [20]. Two intervention groups were established: the PNE + TE group and the TE group. This clinical trial was designed in accordance with the Consolidated Standards of Reporting Trials (CONSORT) Guidelines. The study was approved by the Clinical Research Ethics Committee of Burgos-Soria (CEIC 1903) and registered at clinicaltrials.org (NCT03641495). All patients provided written informed consent prior to enrolment.

### 2.2. Participants

A total of 32 female patients with FMS (mean age: 52.59 ± 10.36) were enrolled in the study, referred from medical doctors and from the fibromyalgia association FIBROAS (Soria, Spain).

To be eligible, participants were required to be women diagnosed with FMS by a rheumatologist in accordance with the 2016 American College of Rheumatology (ACR) classification criteria; they were also required to be aged between 20 and 65 years and to agree to attend therapy sessions.

Patients were excluded if they reported any cardiovascular, respiratory, metabolic, neurological, rheumatic, renal or hepatic, somatic or psychiatric disease or disorder; pregnancy or lactation; changes in pharmacologic therapy in the last three months or during the period of the study; previous exercise or physiotherapy treatments within the last three months; or contraindications or inability to understand the questionnaires.

### 2.3. Randomization and Blinding

After signing the informed consent, the thirty-two patients with FMS were randomly allocated to one of two treatment groups. An external examiner performed the concealed allocation (ratio 1:1) using the Research Randomizer (Version 4.0) computer software. Both the examiner and the patients were blinded to the assigned group.

### 2.4. Interventions

Both groups received the same TE intervention, carried out by a physiotherapist who was an expert in the management of chronic pain and who was blinded to the group allocation. The TE program consisted of 30 sessions (3 sessions of 60 min per week for 10 weeks) and was designed in accordance with the ACSM guidelines.

Each session included an active warm-up based on low-intensity movements and dynamic stretching, a central part with aerobic training and strengthening exercises of the major muscles, and a cooldown part including static stretching and respiratory exercises. The type of exercises and the intensity selected for each were determined in accordance with the protocol of Busch et al. [21].

A detailed description of the intervention is shown in the primary study [20].

All exercises were adapted and controlled by a physiotherapist to ensure patient comfort and safety so as to minimize possible adverse events.

The PNE + TE group also received a face-to-face educational intervention (each session lasting 30–45 min) following the recommendations of Butler and Moseley [22]. This intervention consisted of 8 sessions and was applied by a medical doctor.

The PNE sessions included explanations about acute and chronic pain and the potential sustaining factors of central sensitization such as emotions, stress, illness perceptions, pain cognitions, and pain behavior. Patients were encouraged to apply the new knowledge to their daily life. All questions were answered during the study.

### 2.5. Outcome Measures

Sociodemographic information including gender, age, height, weight, body mass index (BMI), and years since diagnosis were registered for descriptive purposes. The outcome variables were general fatigue, sleep disturbances, physical function, and maximum isometric strength. All the outcome variables were measured at baseline (T0), 1 week after the 10-week intervention (T1), and at 3 months of follow-up (T2), by 3 examiners blinded to treatment allocation. T1 was measured 1 week after completing the intervention to avoid any related fatigue.

### 2.6. General Fatigue and Sleep Disturbances

General fatigue and sleep disturbances were recorded using a 10 cm Visual Analogue Score (VAS) [23,24,25]. The ends represented the extreme expressions, in which 0 represented “no symptoms or disturbances” and 10 “the most intense symptoms or disturbances”. Patients were asked to report fatigue and sleep disturbances in the last 3 days. The VAS-F and the VAS-S have been widely used for assessing general fatigue and sleep disturbances in patients with FMS. The VAS scale has been shown to be valid and reliable for the measurement of fatigue and sleep disturbances in patients with FMS [24]. The minimum clinical difference has been stated to stand at 2 points [25].

### 2.7. Physical Function

Physical function was assessed with the Senior Fitness Test (SFT) battery. The SFT is composed of a set of field-based tests that evaluate cardiorespiratory fitness, speed agility, muscular strength, and flexibility [26]. The SFT has been previously used in patients with FMS and has shown an intraclass correlation coefficient (ICC) from 0.93 to 0.98 [27].

Cardiorespiratory fitness was assessed with the 6-Minute Walk Test (6MWT). This test measures the distance, in meters, that patients can walk in 6 min in a 20 m corridor. The minimal detectable change (MDC) has been stated to be 65.2 m in the FMS population [28].

Speed agility was measured with the Timed Up and Go (TUG) test. This test measures the time, in seconds, that patients need to stand up from a chair, walk 3 m to a cone, return to the chair, and sit down again. The MDC for the TUG test has been stated to stand at 1.60 s [28].

Strength was measured with the 30 s chair-to-stand (CS) test for lower limb strength and the Arm Curl (AC) test for upper limb strength. The CS test records the number of sit–stand–sit cycles that the patient can complete in 30 s. The AC test measures the number of times that the patient can curl a dumbbell (2.3 kg for women) in 30 s. The MDC was determined to be 2.52 repetitions for the CS and 3.16 repetitions for the AC [28]. Each test was performed twice, and the best score was registered for statistical analysis.

Flexibility of the lower limb was measured with the chair sit-and-reach test (CSRT), and upper limb flexibility was measured with the back-scratch test (BST). The CSRT was assessed by having patients in a sitting position and asking them to extend one leg and to bend forward to touch the toes. The BST was assessed by having patients in a standing position and asking them to overlap their middle fingers behind their back. The MDC for the two tests was determined to be 8.66 cm for the CRST and in 7.68 cm for the BTS [28]. Each test was performed twice, and the best score was registered for statistical analysis.

The tests were carried out by alternating upper and lower body tests, with adequate pauses between them to prevent fatigue.

### 2.8. Strength

Maximum isometric handgrip strength was measured using a Jamar hydraulic dynamometer (Sammons Preston, Warrenville, IL, USA). Two trials were performed for each hand following the protocol described by Trampische et al. [29]. To prevent fatigue, a 1 min break between tests was allowed. The highest score was used for statistical analysis. This protocol has been shown to be reliable with an ICC from 0.92 to 0.97, and the MDC was determined to be 4.04 [28].

## 3. Statistical Analysis

SPSS software version 20.0 for Windows was used for statistical analysis (SPSS Inc, Chicago, IL, USA). The statistical analysis was conducted according to intention to treat (ITT). Mean and standard deviations were calculated for quantitative variables. Normal distribution of the variables was analyzed using the Shapiro–Wilk test (*p* > 0.05). Baseline demographic and clinical variables were compared between groups using Student’s *t* test or the Mann–Whitney U test according to the normally distributed data or non-normally distributed data.

A two-way repeated-measures analysis of variance (ANOVA) was performed to investigate differences in outcomes with time (baseline, end of treatment, and 3 months of follow-up) as the within-subjects factor and group (PNE + TE and TE) as the between-subjects factor. A *p*-value < 0.05 was considered statistically significant. The effect size (Cohen’s d) was also calculated, to estimate the magnitude of the within-group differences. The magnitude of the difference was classified as small if the value of Cohen’s d ranged from 0.2 to 0.5, as moderate if it ranged from 0.5 to 0.8, or as large if Cohen’s d was greater than 0.8. Moderate and large magnitudes of effect size were considered indicators of appropriate statistical power.

## 4. Results

**Figure 1:**
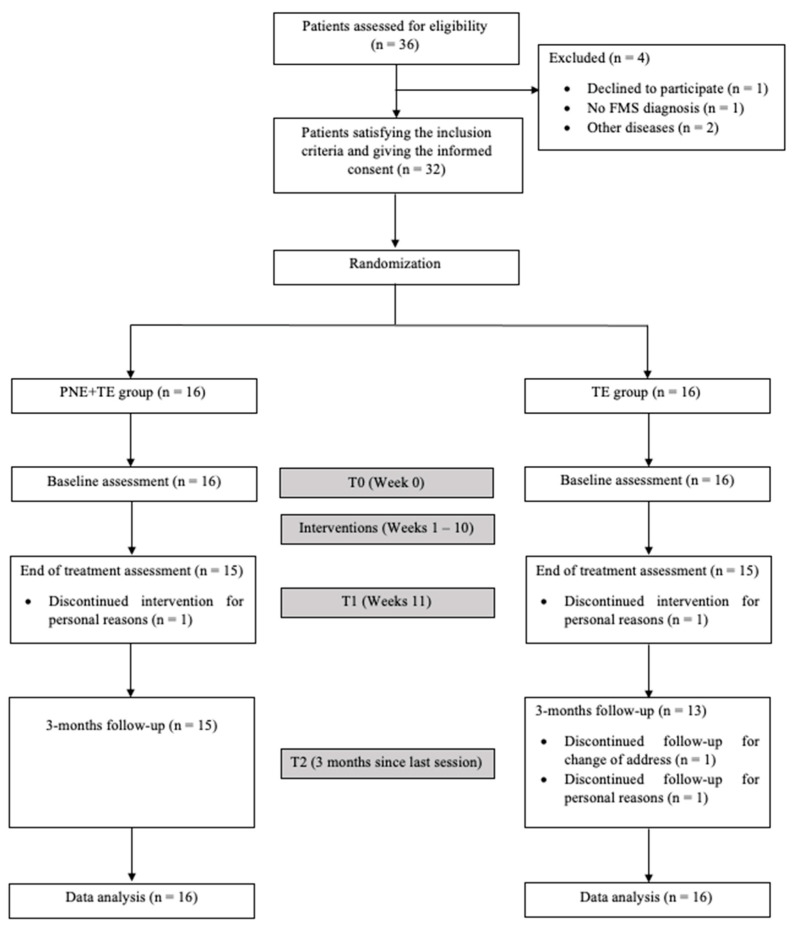
Flowchart diagram.

A total of 32 women with FMS that met the inclusion criteria were randomized into the TE group (*n* = 16) or the PNE + TE group (*n* = 16) (Figure 1). Participants had a mean age of 52.59 (±10.37) years, with a mean height of 1.59 (±0.05) m, mean body weight of 71.13 (±14.9) kg, and mean BMI of 28.02 (±6.09) kg/m^2^. Clinical variables were similar between both groups at baseline (Table 1).

The ANOVA showed a significant group by time interaction after intervention for TUG (F = 4.51; *p =* 0.042; E.S: 0.45) and AC (F = 4.46; *p =* 0.043; E.S: 0.73) at T1 and for handgrip in the non-dominant side (F = 4.86; *p =* 0.036; E.S: 0.33) at T2. The PNE + TE group showed a better performance in the tests compared to the TE group (TUG: ∆ −1.1; 95% CI: −2.87 to −0.67; AC: ∆ 3.46; 95% CI: 0.07 to 6.86; handgrip ND: ∆ 6.63; 95% CI: 0.06 to 13.2). There were no statistically significant differences between groups for the rest of the variables (*p >* 0.05).

The within-group analysis reported that the PNE + TE achieved statistically significant improvements in all the variables at T1, most of which were maintained at T2, except for BST and handgrip in the dominant side (*p >* 0.05). Table 2 provides between- and within-group changes at baseline, after intervention, and at 3 months of follow-up.

## 5. Discussion

This secondary analysis of a randomized controlled trial is the first study to investigate the effects of PNE + TE on fatigue, sleep disturbances, and physical function compared to TE in isolation in patients with FMS.

The baseline data obtained in this study are similar to the cutoff points suggested by Boomershine et al. [30] for fatigue and sleep disturbances and are in agreement with the cutoff proposed by Aparicio et al. [31] for the SFT in patients with FMS. According to this, all patients presented high fatigue levels, severe sleep disturbances, and poor physical function. These baseline results are similar to those of previous studies that included patients with FMS [16,32,33,34]. Cardiorespiratory fitness and muscle strength are the central components of the physical requirements for work and daily life activities and are correlated with pain intensity and sleep disturbances. The results reported in this study show the challenges that women with FMS face when it comes to performing daily life activities. Therefore, these results highlight the importance of considering physical function together with intensity of pain in patients with FMS.

The results of this study showed that the PNE + TE group reported greater improvements on the TUG test and AC test after intervention, and on handgrip at three months of follow-up. No between-groups differences were reported for fatigue, sleep disturbances, or the other tests included in the SFT. The PNE + TE group showed improvements in all variables after intervention and at three months of follow-up except for BST and handgrip in the dominant side. Despite the fact that the improvements achieved did not reach between-groups statistical significance, the changes observed in these variables were higher than the MDCs stated for these tests [28]. The changes shown in the TE group were not higher than the MDCs except for the sleep disturbances and the AC test.

Fatigue is a complex and multidimensional non-pain symptom. Its underlying mechanisms are not clearly known but seem to include some biological patterns, such as cardiac function, chronic inflammatory status, skeletal muscle modifications, nutritional deficiencies, and sleep disturbances [35,36]. Poor sleep quality has been shown to be directly linked to fatigue in patients with FMS [37]. According to this, sleep quality and fatigue are related to several factors that cannot be modified. The application of exercise with or without education appeared to provide some positive effects on these variables. However, not all the factors taken into consideration in the present study may explain the lack of between-groups differences.

Concerning physical function, PNE + TE could modulate short physical function tests and maximum isometric strength. The improvements reported in the TUG, AC, and handgrip tests could be explained by the addition of education to the TE program or by the improvement on pain intensity. Patients with FMS present negative emotions and pain-related fear that have a negative effect on their physical function [38]. PNE tries to normalize attitudes and behavior toward chronic pain using positive coping strategies in dealing with functionality-related complaints [39]. In addition, pain is associated with reduced muscle activation [40]. In the primary study, the patients with FMS who received PNE + TE showed a greater decrease in pain intensity than the TE group [20]. The changes achieved in pain intensity may contribute to better force generation.

The rest of the test included in the SFT did not reach statistical significance between groups but fluctuated somewhat over time. According to this, the addition of eight face-to-face educational sessions to a TE protocol may not contribute to greater benefits in these variables. The use of only education and exercise as well as the duration of the intervention may be insufficient to change all physical function parameters in a multidimensional chronic disease, such as FMS.

From a clinical perspective, the results of the current study reported that the combination of PNE + TE produces greater improvements on the TUG, AC, and handgrip tests than TE in isolation. The addition of education to a TE protocol was shown to be effective in encouraging patients to adopt a more active lifestyle and increase their activity and performance levels [41], which could decrease physical deconditioning in patients with FMS by helping them to avoid sedentary lifestyles and the associated risks of additional morbidity.

This study had several limitations. Firstly, the sample size was calculated based only on the main variable, which may condition the results achieved and make extrapolation of the results difficult. Secondly, the sample was only composed of women, because FMS affects this group much more than men, so the results cannot be extrapolated to men, either. Thirdly, there was no control group, and no binding of patients was practiced, so it is possible that the changes shown in the TE group are not due to the exercise protocol. Fourthly, the duration of the intervention was restricted due to the effort required to manage FMS. Finally, no long-term effects were assessed. Future studies should investigate the long-term effects of PNE + TE in a larger sample size, including men, and with a proper control group.

## 6. Conclusions

PNE + TE was more effective than TE in isolation in improving TUG and AC in the short term and handgrip at three months of follow-up. No between-groups differences were found for fatigue, sleep disturbances, or the other tests included in the SFT after intervention or at three months of follow-up.

## Figures and Tables

**Table 1:** Baseline sociodemographic data.

	PNE + TE Group (*n* = 16)	TE Group (*n* = 16)	*p* Value
Age (years)	52.13 ± 10.31	53.00 ± 10.68	t= 0.218 0.818
Height (cm)	159.66 ± 5.08	159.35 ± 5.87	t = 0.385 0.874
BMI (kg/cm^2^)	30.10 ± 6.69	26.17 ± 4.99	t= 2.154 0.070
VAS-F	7.15 ± 1.68	6.44 ± 2.29	t = 1 *p* = 0.324
VAS-S	7.11 ± 2.82	6.04 ± 2.69	U= 92 *p* = 0.184
TUG	10.18 ± 3.19	9.25 ± 1.83	U = 118.5 *p* = 0.724
6MWT	390.37 ± 105.64	397.06 ± 64.27	U = 117.5 *p* = 0.696
CS	10.06 ± 4.36	8.62 ± 2.22	U = 97.5 *p* = 0.254
AC	10.44 ± 4.27	8.75 ± 3.22	t = 1.262 *p* = 0.217
CSR	−9.00 ± 11.56	−4.37 ± 12.34	U = 87.5 *p* = 0.128
BST	−9.06 ± 11.56	−4.38 ± 12.34	U = 77.5 *p* = 0.056
Handgrip D	26.00 ± 11.07	21.35 ± 5.37	U = 87.5 *p* = 0.128
Handgrip ND	23.31 ± 10.26	20.56 ± 7.42	U = 118.5 *p* = 0.724

Pain neurophysiology education + exercise therapy: PNE + TE; exercise therapy: TE; Visual Analog Scale of Fatigue: VAS-F; Visual Analog Scale of Sleeping disturbances: VAS-S; Timed Up and Go: TUG; 6-Minute Walk Test: 6MWT; chair sit-and-reach test: CSRT; 30 s chair-to-stand: CS; chair sit-and-reach test: CSR; back-scratch test: BST; dominant: D; not dominant: ND.

**Table 2:** Descriptive data, between-groups and within-group statistical significance, and score changes on ITT analysis.

	Baseline (T0) Mean ± SD (95% CI)	Postintervention (T1) Mean ± SD (95% CI)	Within-Group Changes *p*-Values	Between-Groups *p*-Value	3 Months Follow-Up (T2) Mean ± SD (95% CI)	Within-Group Changes *p*-Values	Between-Groups *p*-Value
VAS-F (0–10)							
PNE + TE group	7.15 ± 1.68	5.11 ± 3.81	2.04 (0.48, 3.59) <0.013	F = 0.21 0.648	5.45 ± 2.66	1.70 (0.20, 3.19) <0.028	F = 1.04 0.315
TE group	6.44 ± 2.29	4.78 ± 2.99	1.65 (0.73, 2.57) 0.002		5.6 ± 2.38	0.83 (−0.2, 1.86) 0.107	
VAS-S (0–10)							
PNE + TE group	7.11 ± 2.82	4.71 ± 3.52	2.40 (0.30, 4.50) 0.027	F = 2.68 0.112	5.00 ± 2.95	2.01 (0.03, 3.05) 0.040	F = 1.00 0.324
TE group	6.04 ± 2.69	5.53 ± 2.90	0.51 (−0.76, 1.79) 0.405		3.67 ± 2.57	2.37 (1.40, 3.33) <0.001	
TUG							
PNE + TE group	10.18 ± 3.19	7.53 ± 1.97	2.65 (1.70, 3.50) 0.010	F = 4.51 0.042	7.65 ± 2.16	2.53 (1.69, 3.36) <0.001	F = 3.81 0.060
TE group	9.25 ± 1.83	8.63 ± 2.86	0.62 (−1.17, 2.42) 0.472		7.72 ± 1.87	0.83 (0.83, 2.23) <0.001	
6MWT							
PNE + TE group	390.37 ± 105.64	463.31 ± 120.05	−72.93 (−98.94, −46.91) <0.001	F = 2.63 0.115	435.24 ± 122.73	−44.86 (−76.94, −12.78) 0.009	F = 0.32 0.575
TE group	397.06 ± 64.27	445.71 ± 74.34	−48.65 (−67.04, −30.25) <0.001		432.79 ± 59.53	−35.72 (−48.10, −23.34) <0.001	
CS							
PNE + TE group	10.06 ± 4.36	12.47 ± 4.27	−2.40 (−3.14, −1.67) < 0.001	F = 1.03 0.316	12.26 ± 5.12	−2.20 (−3.13, −1.26) <0.001	F = 0.06 0.803
TE group	8.62 ± 2.22	10.49 ± 2.82	−1.86 (−2.73, −0.99) 0.001		10.69 ± 2.24	−2.06 (−2.75, −1.37) <0.001	
AC							
PNE + TE group	10.44 ± 4.27	14.97 ± 5.02	−4.53 (−6.01, −3.05) <0.001	F = 4.46 0.043	15.75 ± 5.76	−5.31 (−7.34, −3.28) <0.001	F = 2.69 0.111
TE group	8.75 ± 3.22	11.50 ± 4.37	−2.75 (−3.77, −1.73) <0.001		12.38 ± 4.09	−3.62 (−4.44, −2.80) <0.001	
CSR							
PNE + TE group	−9.00 ± 11.56	−5.71 ± 13.59	−9.66 (−17.05, −2.27) 0.014	F = 1.36 0.251	−4.17 ± 15.2	−11.2 (−20.64, −1.75) 0.023	F = 3.31 0.079
TE group	−4.37 ± 12.34	−3.04 ± 9.96	−4.96 (−9.29, −0.62) 0.028		−5.76 ± 13.86	−2.23 (−6.83, 2.37) 0.318	
BST							
PNE + TE group	−9.06 ± 11.56	−8.99 ± 14.20	−0.06 (−4.95, 4.82) 0.977	F = 0.020 0.887	−7.59 ± 13.20	−1.46 (−5.64, 2.71) 0.466	F = 1.09 0.304
TE group	−4.38 ± 12.34	−3.94 ± 12.78	−0.43(−3.02, 2.15) 0.724		−5.2 ± 14.81	0.83 (−1.29, 2.95) 0.427	
Handgrip D							
PNE + TE group	26.00 ± 11.07	29.47 ± 10.07	−3.46 (−7.42, 0.49) 0.082	F = 1.77 0.193	29.40 ± 10.34	−3.46 (−8.04, 1.10) 0.127	F = 0.963 0.334
TE group	21.35 ± 5.37	21.78 ± 8.08	−0.53 (−3.06, 2.00) 0.661		22.28 ± 7.98	−1.03 (−3.66, 1.58) 0.130	
Handgrip ND							
PNE + TE group	23.31 ± 10.26	27.51 ± 9.31	−4.20 (−7.14, −1.21) 0.008	F = 3.6 0.067	27.3 ± 10.35	−3.98 (−7.73, −0.24) 0.038	F = 4.86 0.036
TE group	20.56 ± 7.42	21.63 ± 6.99	−1.06 (−2.99, 0.86) 0.259		20.66 ± 7.63	−0.1 (−0.51, 0.31) 0.611	

Pain neurophysiology education + exercise therapy: PNE + TE; exercise therapy: TE; Visual Analog Scale of Fatigue: VAS-F; Visual Analog Scale of Sleeping disturbances: VAS-S; Timed Up and Go: TUG; 6-Minute Walk Test: 6MWT; chair sit-and-reach test: CSRT; 30 s chair-to-stand: CS; chair sit-and-reach test: CSR; back-scratch test: BST; dominant: D; not dominant: ND.

## Data Availability

This is not applicable.
